# Finite Element Analysis of Platform Switching Effects on Stress Distribution in Posterior Implants Placed in Different Bone Types Under Axial and Oblique Loading Conditions

**DOI:** 10.7759/cureus.86821

**Published:** 2025-06-26

**Authors:** Kanika Yadav, Sandeep Kumar, Rajnish Aggarwal, Iqbal Kaur, Ankit Goyal, Rahul Sharma, Satyendra Banjara

**Affiliations:** 1 Department of Prosthodontics, Surendera Dental College and Research Institute, Sri Ganganagar, IND; 2 Department of Orthodontics, Surendera Dental College and Research Institute, Sri Ganganagar, IND; 3 Department of Oral and Maxillofacial Surgery, Dr. S.S. Tantia Medical College, Hospital and Research Center, Sri Ganganagar, IND

**Keywords:** bone, dental implants, finite element analysis, platform switching, posterior implants, preservation, stresses

## Abstract

Introduction: The present study was conducted to compare stress distribution in platform-switched and non-platform-switched implants placed in D2 (mandible) and D3 (maxilla) bones under axial and oblique loading, using finite element analysis (FEA).

Materials and methods: Cone-beam computed tomography (CBCT)-derived three-dimensional models of the posterior maxilla (D3) and mandible (D2) were developed. Implants (11.5 × 4.2 mm) were modeled with two abutment configurations: 4.2 mm (non-platform switching) and 3.2 mm (platform switching). Porcelain-fused-to-metal crowns were placed on all models. A vertical (axial) load of 200 N and an oblique load of 200 N at 30° were applied to the left first molar. ANSYS Workbench (ANSYS, Inc., Canonsburg, Pennsylvania) was used to assess the von Mises stress distribution in the cortical bone, cancellous bone, implant, abutment, and abutment screws.

Results: Platform switching resulted in lower stress values in both D2 and D3 bones under axial and oblique loads, especially at the crestal bone level. The D3 (maxillary) models exhibited higher stress concentrations overall than the D2 (mandibular) models, indicating a greater biomechanical challenge in less dense bone. Platform switching effectively reduced the peak stresses and led to a more uniform stress distribution. However, the implant and abutment components in the platform-switched models experienced higher internal stress.

Conclusion: Platform switching improved stress distribution and reduced crestal bone stress in both D2 and D3 bones, especially under oblique loading.

## Introduction

Reconstruction of the posterior maxillary and mandibular segments presents unique functional and biomechanical challenges for dental implantology. These regions often undergo substantial bone resorption following tooth extraction, leading to compromised bone volume and density, which can adversely affect both implant stability and long-term success [1,2]. Implant-supported prostheses have become a widely accepted treatment modality for replacing missing posterior teeth, owing to their durability and capacity to restore masticatory function [3]. Adell et al. [4] were the first to document crestal bone resorption in a retrospective study spanning 15 years. Their research revealed a marginal bone reduction of 1-2 mm from the initial thread during the healing phase, as well as in the first year after loading. Following this period, the average annual bone loss of 0.1 mm was observed.

To mitigate marginal bone resorption and enhance aesthetic outcomes, platform switching (PS) has gained considerable attention as a biomechanical strategy to preserve crestal bone levels. PS involves the use of a smaller diameter abutment on a wider diameter implant platform [5]. This configuration is hypothesized to reduce crestal bone stress and minimize marginal bone loss by distancing inflammatory cell infiltrates and redistributing functional loads away from the crestal bone [6]. In contrast, in non-platform switching (NPS), the abutment and implant platform diameters are congruent, which may transmit greater stress concentrations directly to the crestal bone, potentially increasing the risk of bone remodeling or resorption over time [7].

Bone quality is a critical determinant of implant success, particularly in the posterior jaw region where low-density bone is more prevalent. The classification system proposed by Misch and Judy [8] is commonly used to categorize bones based on their density and structure. Bone types D1 and D2, which are found predominantly in the anterior mandible and parts of the posterior mandible, offer better primary stability, whereas bone types D3 and D4, which are more common in the posterior maxilla, are associated with a lower density and reduced implant support. These differences in bone quality can significantly affect stress distribution, implant integration, and clinical outcomes.

The elastic modulus of cortical bone plays a key role in controlling stress concentrations at the crestal interface, which is vital for ensuring implant longevity [9]. Elevated stress and strain in this region can lead to undesirable outcomes, such as bone resorption or implant failure, if not properly managed [10]. Implants placed in areas with diminished bone density, particularly the posterior maxilla, face an increased risk of biomechanical complications owing to higher stress concentrations and fatigue-induced failures [11].

Although finite element analysis (FEA) has been extensively used to assess stress distribution around dental implants, much of the existing literature has focused on implant positioning and angulations. There are comparatively limited data evaluating the biomechanical impact of PS versus NPS under varied bone densities and loading conditions [12,13]. Additionally, there is limited literature exploring how different loading directions, especially oblique forces that simulate masticatory dynamics, affect stress patterns in the posterior maxilla and mandible [14,15]. Oblique loading, particularly at angles that replicate functional occlusion, provides clinically relevant insights into real-life biomechanical challenges [16].

This in vitro FEA study aimed to evaluate stress distribution in bone-surrounding platform-switched and non-platform-switched implants placed in the posterior maxilla and mandible. By incorporating axial and oblique loading conditions across varying bone qualities, this study aimed to enhance the understanding of how implant-abutment configurations could influence stress patterns.

## Materials and methods

Digital model design

Using cone-beam computed tomography (CBCT) scans obtained from the institutional database, anatomically accurate three-dimensional (3D) digital models of the posterior maxillary and mandibular regions were created using CATIA V5 software (Dassault Systèmes, Vélizy-Villacoublay, France). As CBCT scans were obtained from the database, Institutional Ethical Committee clearance was waived for this study (SDCRI/IEC/23/66), and written informed consent was obtained from the patients to use their records for study purposes, maintaining confidentiality. The study was conducted in the time span of six months from October 2023 to March 2024.

These models reflect two bone types based on clinical classification [8], with the posterior maxilla approximating the D3 bone and the posterior mandible resembling the D2 type bone. The bone blocks were uniformly dimensioned at 14 mm height, 8 mm mesiodistally, and 8 mm buccolingually. For the maxillary bone, the thickness of the cortical bone was determined to be 3.6 mm, with a cancellous bone core measuring 4.4 mm. Specifically, the thickness of the palatal and buccal cortical bones was 1.98 and 1.62 mm, respectively. Similarly, the mandibular bone was modeled for the posterior mandibular area, featuring a cancellous bone core thickness of 3.2 mm and a cortical bone thickness of 4.8 mm. The lingual and buccal cortical bone thicknesses in this area were 2.54 and 2.26 mm, respectively [17].

Implant and abutment modeling

A titanium dental implant was digitally modeled using specifications derived from the Adin Internal-Hex implant system (Touareg^TM^-S, Adin Dental Implants, Afula, Israel), which is known for its superior thread design and mechanical performance. The implant measured 11.5 mm in length and 4.2 mm in diameter, with detailed geometric features, including a collar height of 1.5 mm, a thread pitch of 1.2 mm, a thread height of 0.7 mm, and a 2 mm tip diameter. Four maxillary and four mandibular models were processed by using two different abutments of 4.2 mm and 3.2 mm diameter abutment connections, assuming an NPS and PS configuration, respectively. To replicate the prosthetic apparatus, a porcelain-fused-to-metal (PFM) crown was fabricated for the left maxillary or mandibular first molar, measuring 7.0 mm in height and 8.0 mm in buccolingual width, and a corresponding model of this crown was likewise produced. The PFM crown was designed and positioned on the abutment to replicate the prosthetic loading conditions.

Finite element mesh generation and boundary conditions

Following digital modeling, a finite element mesh was generated using the ANSYS Workbench (ANSYS, Inc., Canonsburg, Pennsylvania), which specializes in advanced FEA. The models were discretized into fine tetrahedral elements to ensure computational accuracy. Mesh convergence analysis was performed to optimize the element size, focusing particularly on critical stress concentration areas, such as the implant-bone interface. Convergence was confirmed when further refinement produced less than 5% variation in the peak stress values.

Precise boundary conditions were set to simulate realistic clinical scenarios. The bone block's base was fully fixed in all directions (X, Y, Z axes) to mimic the maxillary bone's anchorage within the skull, preventing displacement during masticatory forces and replicating the restrictive craniofacial environment. All components, implants, abutments, bones, and prostheses were modeled with perfect bonding, particularly at the implant-bone interface, assuming complete osseointegration. This eliminated micromovement or interfacial slip, enabling accurate force transmission analysis. No frictional or contact gaps were included at the interfaces to simplify the computations and to focus on the biomechanical behavior of the implant system.

Material properties and contact assumptions

Material properties are defined based on established biomechanical data [18-20]. All materials, including the cortical bone, trabecular bone, titanium implant, and titanium abutment, are believed to be isotopic, homogeneous, elastic, and linear. The material properties are listed in Table 1.

**Table 1:** Material properties used in the study. Ni-Cr: nickel-chromium; GPa: gigapascals.

Material	Jaw type	Young's modulus (GPa)	Poisson's ratio
Cortical bone [18]	Maxilla	13.7	0.30
	Mandible	13.7	0.30
Cancellous bone [19]	Maxilla	1.6	0.30
	Mandible	5.5	0.30
Titanium [18]		110	0.35
Mucosa [19]		10	0.40
Ni-Cr [20]		203.6	0.30

Loading conditions

A vertical (axial) force of 200 N was applied along the long axis of the implant to simulate the normal chewing forces. In addition, an oblique force of 200 N was applied at an angle of 30°. These values are widely accepted in the literature as representative of functional occlusal forces in the molar and premolar areas [21]. The 200 N vertical load simulated axial forces from normal mastication, while the 30° oblique load mimicked lateral and off-axis forces during functional movements, such as grinding and chewing, offering a clinically relevant evaluation of stress distribution.

Model variants and outcome analysis

Four maxillary and four mandibular models were processed using two abutment diameters. FEA focused on two primary biomechanical outcomes: von Mises stress distribution and strain patterns on the cortical bone, cancellous bone, implant, abutment, and abutment screw.

## Results

**Figure 1:**
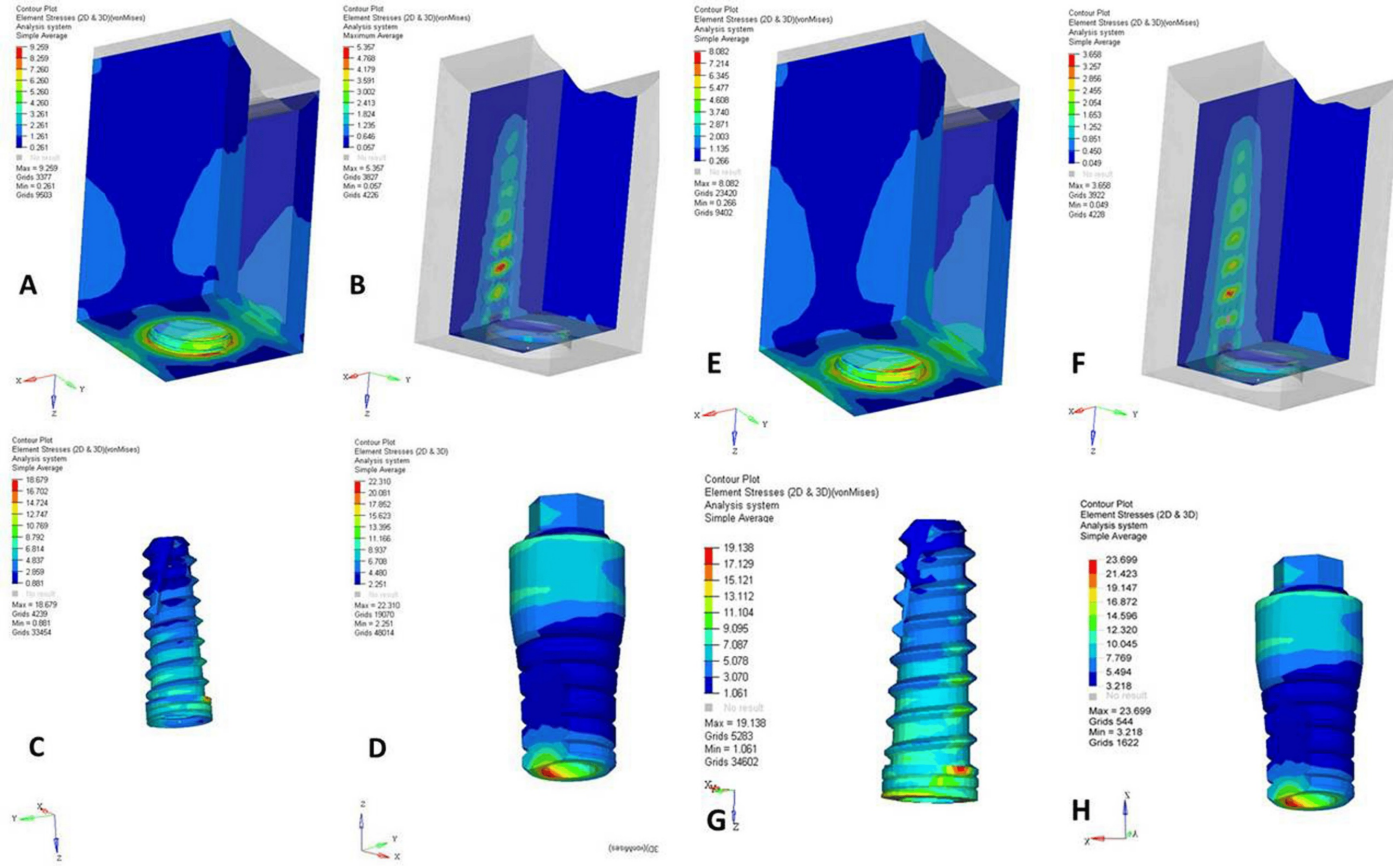
von Mises stresses in maxillary non-platform-switched (NPS) and platform-switched (PS) models under 200 N axial loading. (A) Cortical bone (NPS), (B) cancellous bone (NPS), (C) implant (NPS), (D) abutment (NPS), (E) cortical bone (PS), (F) cancellous bone (PS), (G) implant (PS), and (H) abutment (PS). The figure is derived directly from finite element software based on data from the study.

**Figure 2:**
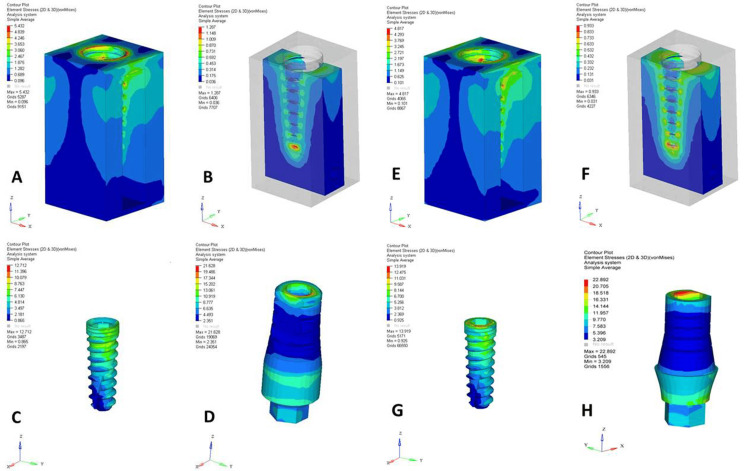
von Mises stresses in mandibular non-platform-switched (NPS) and platform-switched (PS) models under 200 N axial loading. (A) Cortical bone (NPS), (B) cancellous bone (NPS), (C) implant (NPS), (D) abutment (NPS), (E) cortical bone (PS), (F) cancellous bone (PS), (G) implant (PS), and (H) abutment (PS). The figure is derived directly from finite element software based on data from the study.

**Figure 3:**
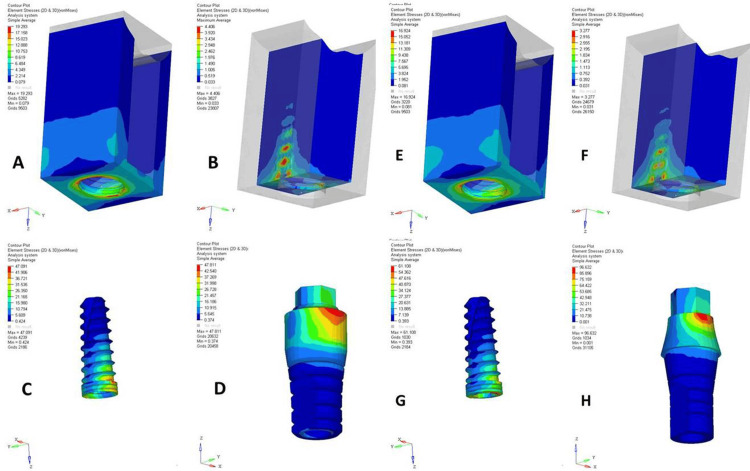
von Mises stresses in maxillary non-platform-switched (NPS) and platform-switched (PS) models under 200 N oblique loading. (A) Cortical bone (NPS), (B) cancellous bone (NPS), (C) implant (NPS), (D) abutment (NPS), (E) cortical bone (PS), (F) cancellous bone (PS), (G) implant (PS), and (H) abutment (PS). The figure is derived directly from finite element software based on data from the study.

**Figure 4:**
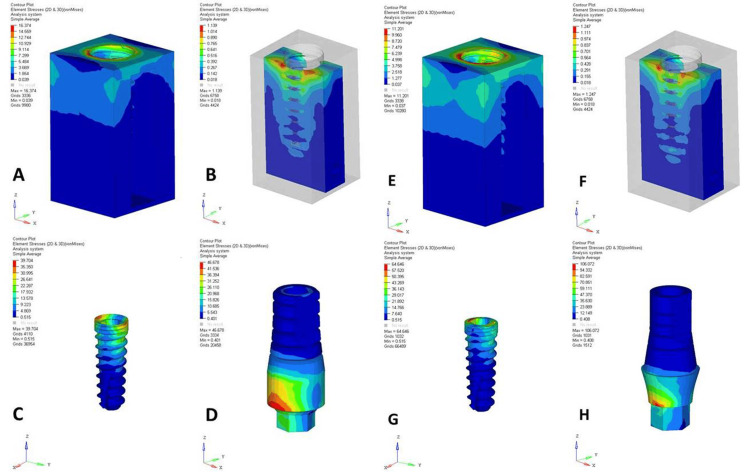
von Mises stresses in mandibular non-platform-switched (NPS) and platform-switched (PS) models under 200 N oblique loading. (A) Cortical bone (NPS), (B) cancellous bone (NPS), (C) implant (NPS), (D) abutment (NPS), (E) cortical bone (PS), (F) cancellous bone (PS), (G) implant (PS), and (H) abutment (PS). The figure is derived directly from finite element software based on data from the study.

A comparative analysis of the NPS and PS dental implant designs under axial (Figures 1, 2) and oblique loadings (Figures 3, 4) in the maxillary and mandibular arches revealed distinct stress distribution patterns in the cortical and cancellous bone, abutments, implants, and abutment screws. Under axial loading, the NPS model exhibited higher von Mises stress concentrations near the crestal region of cancellous bone, potentially increasing the risk of bone microdamage or resorption. In contrast, the PS model demonstrated a more uniform and lower stress distribution throughout the cancellous bone, suggesting better load distribution. The stress in the NPS design was more pronounced in the coronal region of the abutment, particularly at the implant-abutment junction, increasing the risk of mechanical failure. However, the PS model exhibited a more evenly distributed stress pattern, reducing the stress concentration.

Under oblique loading, the NPS model displayed higher stress concentrations in both the cortical and cancellous bones near the implant-bone interface, especially at the crestal bone. The PS model exhibited a more favorable stress distribution, with reduced stress levels near the crestal bone and a more dispersed pattern. In the NPS design, stress was concentrated at the abutment-implant junction, potentially leading to mechanical complications such as micro-movements or fatigue failure. Conversely, the PS model showed dispersed stress at the junction, indicating better load transfer and enhanced implant-abutment interface longevity. The NPS model also had higher stress in the crestal and coronal regions of the implant, risking mechanical overload, whereas the PS model distributed stress more evenly along the implant body.

Quantitative data from a 200 N axial load (Table 2) showed that PS consistently reduced the maximum stress values across all structures compared to NPS. The cortical and cancellous bones in the PS models exhibited lower stresses, with the maxillary jaw experiencing higher stresses. However, abutments, implants, and screws in the PS models showed higher peak stresses, particularly in the maxilla, indicating a shift in stress to implant components. Under a 200 N oblique load (Table 3), cortical bone stresses were higher in the NPS, with the mandible showing lower stresses. The cancellous bone showed a similar trend. Implants in the PS models experienced higher stresses, possibly due to altered stress transfer. Overall, PS reduced bone stress more effectively, especially in the mandible, but increased the stress in the implant components. Oblique loading consistently produced higher von Mises stresses than axial loading across all structures.

**Table 2:** von Mises stresses in megapascals (MPa) resulted under axial load (200 N) in non-platform-switched (NPS) and platform-switched (PS) models in both jaws. Min: minimum; Max: maximum.

Structures	Maxillary jaw		Mandibular jaw	
	NPS	PS	NPS	PS
Cortical bone	Max- 9.259	Max- 8.082	Max- 5.432	Max- 4.817
	Min- 0.261	Min- 0.266	Min- 0.096	Min- 0.101
Cancellous bone	Max- 5.357	Max- 3.658	Max- 1.287	Max- 0.933
	Min- 0.057	Min- 0.049	Min- 0.031	Min- 0.036
Implant	Max- 18.679	Max- 19.138	Max- 12.712	Max- 13.919
	Min- 0.881	Min- 1.061	Min- 0.865	Min- 0.925
Abutment	Max- 22.310	Max- 23.699	Max- 21.628	Max- 22.892
	Min- 2.251	Min- 3.218	Min- 2.351	Min- 3.209
Abutment screw	Max- 6.720	Max- 9.599	Max- 9.075	Max- 6.500
	Min- 1.790	Min- 2.149	Min- 2.070	Min- 1.791

**Table 3:** von Mises stresses in megapascals (MPa) resulted under oblique load (200 N) in non-platform-switched (NPS) and platform-switched (PS) models in both jaws. Min: minimum; Max: maximum.

Structures under loading	Maxillary		Mandibular	
	NPS	PS	NPS	PS
Cortical bone	Max- 19.293	Max- 16.924	Max- 16.374	Max- 11.201
	Min- 0.079	Min- 0.081	Min- 0.039	Min- 0.037
Cancellous bone	Max- 4.406	Max- 3.271	Max- 1.247	Max- 1.139
	Min- 0.033	Min- 0.031	Min- 0.018	Min- 0.018
Implant	Max- 47.091	Max- 61.108	Max- 39.704	Max- 64.646
	Min- 0.424	Min- 0.393	Min- 0.515	Min- 0.515
Abutment	Max- 47.811	Max- 96.632	Max- 46.678	Max- 106.072
	Min- 0.374	Min- 0.001	Min- 0.401	Min- 0.408
Abutment screw	Max- 6.720	Max- 9.599	Max- 6.500	Max- 9.075
	Min- 1.790	Min- 2.149	Min- 1.791	Min- 2.070

## Discussion

PS has been used to mitigate bone resorption in the vicinity of dental implants, thereby enhancing their longevity [5]. It involves using an abutment with a smaller diameter than the implant and shifting the implant-abutment interface from the implant's periphery to its central axis. This design reduces compressive forces and mechanical stress on the surrounding bone, particularly near the crestal region, promoting better stress distribution and bone preservation [5-7].

Under axial loading conditions, von Mises stress analysis revealed distinct differences in stress localization between the PS and NPS models. In the NPS design, stress concentrations were highly localized and intensified around the crestal region of the cancellous bone and the coronal portion of the implant-abutment interface. Such stress accumulation near the crestal bone is clinically concerning as it may contribute to bone microdamage and resorption, jeopardizing implant stability over time.

Conversely, the PS design demonstrated a more uniform and lower-magnitude stress distribution across both cortical and cancellous bones. This finding suggests that inward repositioning of the abutment during PS may redirect stresses away from the vulnerable crestal bone toward the central portion of the implant. The more favorable stress pattern observed in the PS design supports its role in reducing crestal bone loss, a common complication that can compromise the esthetics and functionality of implant-supported restorations.

Crestal bone resorption around dental implants, typically 1.5 mm in the first year and 0.2 mm annually thereafter, is considered an acceptable standard for two-piece dental implant systems [22]. However, maintenance of crestal bone levels is crucial for implant success. PS connections have the potential to mitigate marginal bone resorption by facilitating horizontal inward displacement of the implant-abutment connection. On meticulous histological examination of PS implants, Sasada and Cochran [23] elucidated that the interface between the implant and abutment (microgap) is enveloped by the connective tissue. Empirical clinical investigations have also indicated that reduced bone resorption is observed when employing a narrower-diameter abutment in conjunction with the PS technique [5-7]. PS induces lateral migration of the inflammatory cell infiltrate away from the crestal bone, thus augmenting the spatial separation between the implant-abutment junction and the crestal bone level [24]. A previous systematic review has concluded that PS leads to less crestal bone loss [25]. In the PS models, the stress distribution around the implant-abutment junction was more balanced but showed higher peak stress values compared to NPS. This is likely due to the larger abutment diameter in NPS, which enhances stress distribution over a larger contact area between the abutment, implant, and prosthesis.

The PS uses a narrower abutment on a wider implant, creating a horizontal offset. Under oblique loading, this offset acted as a longer lever arm, thereby increasing the bending moment at the screw interface. Consequently, lateral forces increase, causing higher stress on the abutment screw in PS implants compared to NPS implants during off-axis loading. According to a study by Segundo et al. [26], the internal-hex system leads to a higher stress concentration around the implant neck and prevents the accumulation of stress on the abutment screw, as observed in our study. Mitra et al. [16] also reported increased stress concentration at the neck of PS implants in the internal-hex system. Therefore, the design of the PS internal-hex implant with its narrower abutment and internal connection geometry results in a mechanical disadvantage at the implant neck by concentrating the functional stresses in that region, particularly under lateral or complex loading conditions.

This could potentially maximize the risk of mechanical failures, such as screw loosening, abutment fracture, or implant-abutment micro-movements. In contrast, the NPS design exhibited a concentrated stress peak in the coronal part of the abutment, suggesting higher susceptibility to mechanical complications in these regions. This finding is in accordance with a study by Sahabi et al. [27], who found more stress in implants and abutment screws in PS than in NPS.

This study found higher stresses on implant components in PS models, with variations between arches. In the maxillary arch, higher stresses under axial loading result from lower bone density and a thinner cortical layer, forcing implant components to bear more load. In the mandibular arch, higher stresses under oblique loading stemmed from denser and more rigid bone, which transferred greater shear and bending forces to the implant components. PS redistributed stress toward implant components, amplifying these effects, with the maxilla experiencing higher stresses under axial loading and the mandible under oblique loading owing to their distinct biomechanical properties. However, in accordance with the research investigation conducted by Maeda et al. [28], such complications manifested solely when the applied stresses surpassed the elastic threshold. The observed escalations in stress magnitudes may not precipitate any significant complications, given that the yield strength of titanium alloy ranges from 620 to 725 MPa, whereas that of nickel-chromium alloy ranges from 415 to 620 MPa [29].

Oblique loading represents a more clinically relevant simulation of masticatory forces owing to the multidirectional nature of functional occlusal forces. Oblique loading led to increased stress in both PS and NPS. Under these conditions, both the maxillary and mandibular arches exhibited elevated von Mises stress values, particularly in NPS models. The crestal bone again emerged as a critical site for stress concentration in NPS designs, reinforcing concerns regarding potential bone resorption and early implant failure under oblique forces [25].

The PS design effectively redistributed oblique stresses with significantly reduced stress values near the crestal bone and more dispersed patterns across the implant structure. This stress attenuation effect is crucial for preserving the marginal bone levels and maintaining implant health over time. The even distribution of oblique stresses in PS designs likely contributes to improved osseointegration and a reduced incidence of peri-implantitis, as lower stress concentrations minimize the risk of bone overload and subsequent inflammatory responses [24,25].

An interesting finding of this study was the variation in the stress distribution between the maxillary and mandibular arches. Across both loading scenarios, the maxillary arch exhibited consistently higher stress values than the mandible for all structural components. For instance, under axial loading, the cortical bone in the maxilla reached a stress peak of 9.259 MPa in the NPS model compared with 5.432 MPa in the mandible. This discrepancy may be attributed to anatomical and density differences between the maxillary and mandibular bones, with the maxilla generally having less dense and more trabecular bone, which may be more susceptible to stress accumulation. Similar findings were reported by Arun Kumar et al. [18], who reported higher stresses in D3 bone than in D2 bone owing to the presence of more low-density trabecular bone in D3 [8], which is unable to withstand high stresses.

Furthermore, in our study, stress was higher in the cortical bone than in the cancellous bone in all models. This phenomenon can be attributed to variations in the modulus of elasticity between cortical and cancellous bones. Cortical bone, characterized by a higher modulus of elasticity, exhibits greater resistance to deformation and is capable of supporting more load than cancellous bone. Additionally, the elevated stress concentration in the cortical bone can be attributed to the fact that the mechanical stress distribution predominantly occurs at the bone interface with the implant. The extent of contact between the implant and bone was directly correlated with bone density. Notably, the proportion of bone contact is considerably higher in cortical bone than in cancellous bone [8].

Clinical implications

PS effectively reduced crestal bone stress and promoted favorable stress distribution, potentially improving implant survival and peri-implant bone preservation. It is particularly advantageous in the maxillary arch, where anatomical variations and lower bone quality intensify stress concentration. However, the increased stress on implant components, especially abutments, necessitates the use of more durable abutment materials or designs to withstand higher internal stresses. Careful case selection, precise surgical techniques, and strategic prosthetic planning are essential to maximize the benefits of PS while minimizing the mechanical risks.

Limitations

This study had certain limitations. This study was based on the FEA method or in vitro modeling, which might not fully replicate the complex biological and biomechanical environment in vivo. The material properties were assumed to be homogeneous and isotropic, which may not reflect actual bone variability. The study did not assess fatigue failure or long-term cyclic loading, which could affect the mechanical behavior of the implant-abutment system. While bone stress was reduced, the increased stress on abutments in PS designs required further investigation into clinical failure modes and prosthetic complications.

## Conclusions

This FEA study showed that PS significantly reduced stress concentration in the peri-implant crestal bone compared to NPS, especially under oblique loading. Biomechanical benefits were more evident in the maxillary arch (D3 bone), where lower bone density led to higher stress values than in the mandibular arch (D2 bone). While PS effectively lowered stress in the cortical and cancellous bone, it increased stress in the implant and abutment, particularly at the implant-abutment junction. These results indicate that PS enhanced marginal bone preservation and implant longevity, particularly in lower-density bone and complex loading conditions. However, the increased stress on prosthetic components highlights the need for precise restorative design and robust material selection.
